# A 12-week cluster randomized controlled trial of the effectiveness of an AI-aided DICE algorithm for BPSD management in low-resource settings: a study protocol

**DOI:** 10.3389/fpsyt.2025.1548638

**Published:** 2025-05-23

**Authors:** Yaonan Zheng, Xingyu Zhang, Wenxiu Li, Jun Ji, Huali Wang

**Affiliations:** ^1^ Dementia Care and Research Center, Peking University Institute of Mental Health (Sixth Hospital), Beijing, China; ^2^ National Clinical Research Center for Mental Disorders (Peking University), NHC Key Laboratory for Mental Health, Beijing, China; ^3^ Beijing Haidian Psychological Rehabilitation Hospital, Beijing, China; ^4^ College of Computer Science and Technology, Qingdao University, Qingdao, Shandong, China

**Keywords:** neuropsychiatric syndromes, dice algorithm, AI-aided decision process, Alzheimer disease, dementia

## Abstract

**Background:**

The “Describe-Investigate-Create-Evaluate” (DICE) approach has been considered a guide for managing behavioral and psychological symptoms of dementia (BPSD). However, limited resources may limit the implementation of the DICE approach. With the development of AI technology, the effectiveness of the AI-aided DICE algorithm for BPSD management has yet to be determined. Therefore, this study aims to examine the effectiveness of the AI-aided DICE algorithm for managing BPSD in low-resource settings.

**Methods:**

The cluster randomized controlled trial will be conducted in 12 medical facilities where geriatric psychiatrists are not fully installed. One hundred eighty-four persons with mild and moderate BPSD will be enrolled and randomized to the AI-aided DICE group (n=92) and usual care group (n=92). In the AI-aided DICE group, all participants will receive a comprehensive assessment on a digital triage platform to identify individualized needs and target symptoms, be prescribed a personalized management plan based on the AI-aided decision process, be monitor the implementation of the management plan, and receive follow-up assessment to evaluate the effectiveness. The neuropsychiatric inventory questionnaire and caregiver burden inventory will measure primary and secondary outcomes. The study duration for each participant will be 12 weeks.

**Discussion:**

The study will examine the effectiveness of the AI-aided DICE algorithm for managing BPSD in low-resource settings. The findings will support the implementation of an AI-aided algorithm and leverage the practice of quality care for dementia.

## Background and rationale

According to the latest data from the World Health Organization (WHO), there are currently over 55 million people worldwide who have dementia, and this number is projected to exceed 139 million by 2050 (1). Dementia has become a global public health priority, among which Alzheimer’s Disease (AD) is the most common. AD patients, not only experience cognitive impairments but also exhibit behavioral and psychological symptoms of dementia (BPSD) in different stages of the disease, affecting their social functioning and causing psychological distress to patients, their families, and caregivers, thereby impacting their quality of life (2). The International Psychogeriatric Association (IPA) defined BPSD as “a syndrome comprising diverse psychological reactions, psychiatric symptoms, and challenging behavioral manifestations that accompany cognitive impairment caused by various etiologies (3). BPSD often becomes a primary reason for AD patients to receive hospitalization, increasing the burden on healthcare and caregiving (2). Therefore, effectively treating BPSD is a key and challenging aspect of clinical work in geriatric psychiatry.

Studies have found that atypical antipsychotic drugs, such as risperidone, quetiapine, and olanzapine, are commonly used in clinical practice for the treatment of BPSD (4). Besides drugs, non-pharmacological intervention is always the preferred first-line treatment approach, as many guidelines, medical organizations, and expert groups recommend (5). Due to the complex and diverse causes of BPSD, as well as the variability of symptoms influenced by patients’ cultural level, economic foundation, and caregiving situation, there is currently no “universal” treatment plan that takes into account all factors. Therefore, it is essential to carry out patient-centered individualized interventions. The international expert consensus has summarized the “Describe-Investigate-Create-Evaluate” (DICE) process as a guide for BPSD treatment and management (4, 5), which is also recommended by the expert consensus in China (6). The DICE generally approach involves: (1) Describing the behavior in detail, including antecedents and consequences; (2) Investigating potential medical, environmental, or caregiver-related contributors; (3) Creating a tailored treatment plan prioritizing nonpharmacologic strategies; and (4) Evaluating the effectiveness of interventions and adjust as needed (5). Researchers have found that protocols similar to DICE are helpful in providing personalized psychosocial interventions for patients in the process of exploring cognitive care service systems (7). Long-term psychosocial interventions can alleviate patients’ behavioral problems to a certain extent and improve their quality of life (8). Studies have also found that standardized BPSD intervention can significantly reduce the use of antipsychotic medications in dementia patients and improve clinical outcomes (9, 10). Therefore, promoting the application of the DICE process in clinical practice may be a key strategy to enhance the effectiveness of BPSD interventions.

In psychiatric hospitals and long-term care facilities for dementia patients in China, the development and implementation of psychosocial interventions are not satisfactory (11). Additionally, the majority of dementia patients receive home-based care (12), requiring guidance from community-level healthcare workers in managing BPSD. However, there is a shortage of healthcare professionals at the community level, with limited experience in diagnosing and treating BPSD. This is particularly true for doctors with limited clinical experience, who may face challenges in developing personalized intervention plans, effectively implementing, and executing intervention plans, and dynamically adjusting treatment plans. These challenges have an impact on the effectiveness of interventions for dementia patients. Therefore, there is an urgent need to optimize the DICE process, enhance its practicality in application, and train personnel at all levels of healthcare institutions in BPSD interventions.

A detailed description of BPSD is a crucial step in the DICE process. However, many clinicians face difficulties in describing the symptoms. Previous research has found that conducting comprehensive assessments for patients can greatly help treatment providers understand their situation holistically, acknowledge their value, respect their feelings, and listen to their needs, providing a foundation for developing subsequent psychosocial intervention plans (13). Therefore, based on domestic and international research, we selected an assessment package centered around common BPSD symptoms such as agitation and impulsivity, psychotic symptoms, sleep disorders, and mood disturbances, based on the Neuropsychiatric Inventory (NPI), to assist in identifying the target symptoms in patients and provide detailed descriptions. Furthermore, the occurrence of BPSD is not only related to biological factors associated with the pathogenesis of Alzheimer’s disease but also closely related to psychological, interpersonal, and social-environmental factors. At the investigate step of the DICE, in order to help clinicians in quickly to identify triggers for BPSD and know the patient’s individual condition, we have developed an investigation framework that includes social support, significant life events, physical condition, interests and hobbies, and personality traits which based on a behavioral analysis model (6). In BPSD interventions, the appropriate selection of non-pharmacological interventions could directly decrease stress. When the patient’s stress is improved, BPSD can be significantly alleviated (13). Therefore, we have developed a set of BPSD psychosocial intervention indicators suitable for the Chinese cultural environment and a practical toolkit for caregivers to use at home (14).

Based on this, our team has completed the localization adaptation of the DICE process, forming an artificial intelligence-aided “Describe-Investigate-Create-Evaluate” algorithm (AI-aided DICE algorithm). Whether applying the Optimized DICE Approach in medical institutions can effectively alleviate BPSD and reduce caregiver burden remains unanswered.

Therefore, this study protocol aims to clarify the effectiveness of the Optimized DICE Approach in improving patient clinical outcomes, including alleviating behavioral symptoms, and reducing caregiver burden. The implementation of this project is expected to provide technical support for clinical interventions in BPSD and essential means for achieving comprehensive management of dementia patients. This study employed a cluster randomized controlled trial (RCT) design to evaluate the effectiveness of using the AI-aided DICE algorithm to intervene in the psychiatric behavior symptoms of AD patients and to alter the burden on caregivers. The research hypothesis is that, compared with regular treatment, the intervention group using the AI-aided DICE algorithm can effectively alleviate the severity of BPSD in AD patients and reduce the burden on caregivers.

## Methods and analysis

### Study design

The study design is 12-week, parallel groups, double-blind cluster-randomized control trials (CRCT). The trial protocol has been registered at the Chinese Clinical Trial Registry (Registration number: ChiCTR2400084588). In this trial, 12 medical institutions, including two tertiary hospitals, two secondary hospitals, and eight community health service centers, will be selected for the study. The randomization unit is the medical institution, and medical institutions of the same level will be randomly assigned in a 1:1 ratio to the optimized DICE intervention group and the control group. Each group will include one tertiary hospital, one secondary hospital, and four community health service centers. A total of 184 participants will be included in this study, with 92 participants in each group.

The design includes four assessment time points: baseline, one, four, and 12 weeks. The CRCT consists of a 1-week screening period and a 12-week intervention follow-up period for each participant, with the study being completed within four years.

### Setting

Recruitment of participants for the trial will commence in January 2025 and is scheduled to be completed in November 2026 under the auspices of the Dementia Care and Research Center (DCRC) at Peking University Institute of Mental Health (Sixth Hospital). DCRC has long been committed to providing standardized diagnosis and treatment for patients with cognitive disorders, practical support for carers, and diagnostic support for more than 200 newly referred patients with cognitive impairment every year. The Capital Clinical Characteristic Diagnosis and Treatment Technology Research and Transformation project of the Beijing Municipal Science and Technology Commission funds the research program.

### Participants

Inclusion criteria for participants are: (A) Clinically diagnosed as dementia according to International Classification of Diseases (ICD-10) criteria and classified as Alzheimer’s disease subtype; (B) Age between 60 to 85 years old; (C) The severity score of any item in the Neuropsychiatric Inventory Questionnaire-Informant Version (NPI-Q) was ≥ 2; (D) Having a regular caregiver; (E) Patient or their legal guardian consents to participate in a 12-week follow-up. All study participants receive clinical diagnosis of Alzheimer’s disease (AD) from memory specialists, neurologists, or geriatric psychiatrists in tertiary hospitals. Clinicians make the diagnosis with standard clinical workflow, including collecting clinical information such as onset and progression of symptoms, reviewing medical history, conducting cognitive assessments, and performing laboratory investigations and brain imaging. The investigators of the present proposed study will collect the diagnostic information provided by the tertiary hospitals.

Exclusions criteria include: (A) Clinically diagnosed with types of dementia other than Alzheimer’s disease; (B) With impaired consciousness; (C) Those whose impulsive behaviors are dominated by hallucinations (score of 3 on the hallucination item of NPI-Q); (D) Presenting with extremely severe behavioral and psychological symptoms (score of 3 on the agitation/aggression item of NPI-Q) requiring emergency antipsychotic treatment; (E) With severe physical condition or unstable malignant tumor condition.

Withdrawal from the trial occurs if: (A) The participant is deemed unfit to continue the study for medical reasons; (B) The poor adherence of the participants to the protocol will have a significant impact on the evaluation of the therapeutic effect; (C) The participant requests to withdraw from the study; (D) Participants are lost during follow-up; (E) Researchers assess participants as having a significant risk of harm to themselves or others or NPI-Q agitation/aggression item score =3, and caregiver distress score ≥4 for this item. Withdrawal from the research can be at the request of the participant, carers or the investigator’s discretion.

### Enrolment and randomization

**Figure 1:**
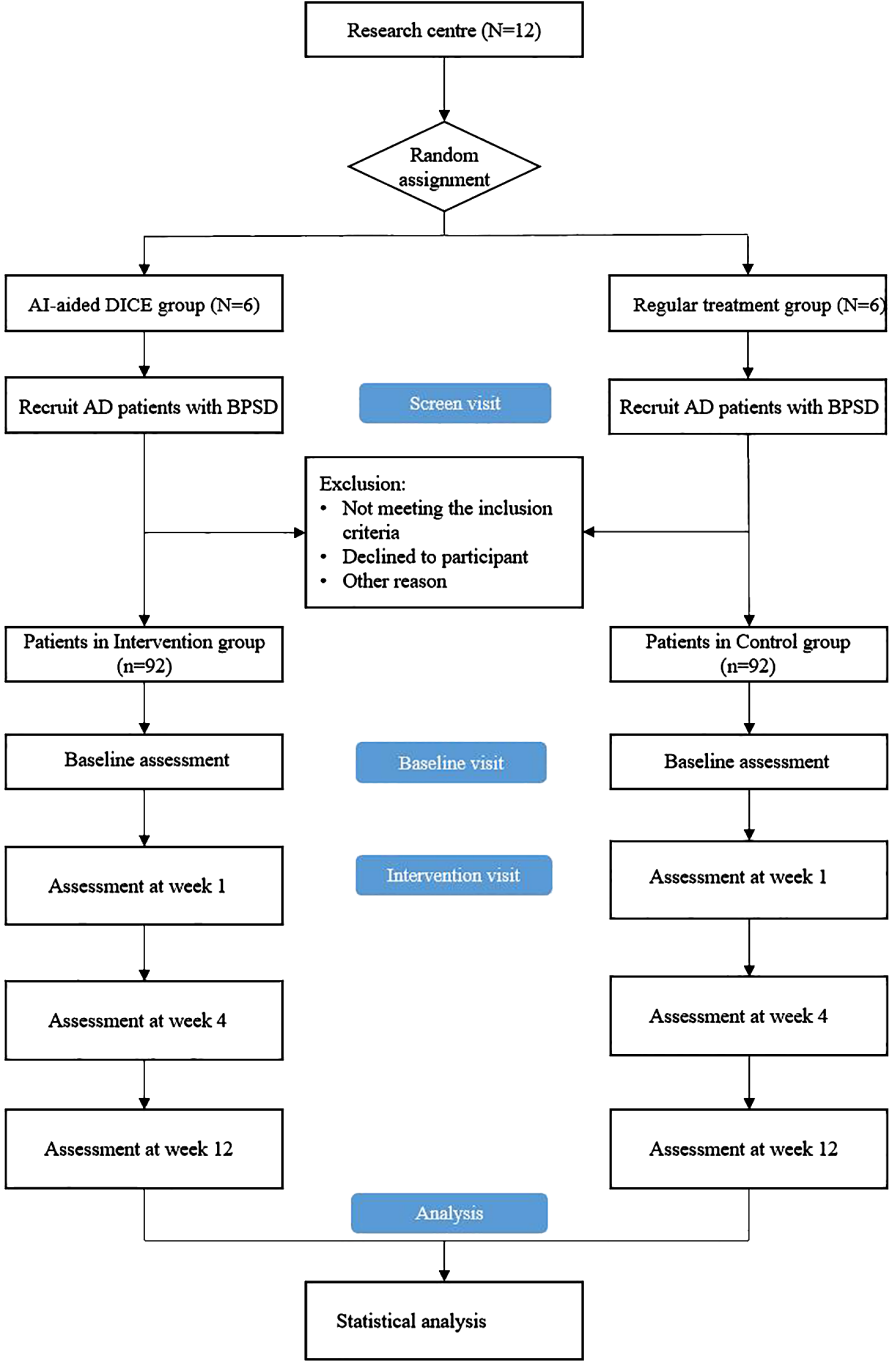
Overview of study procedure. DICE, Describe-Investigate-Create-Evaluate; AD, Alzheimer’s disease; BPSD, behavioral and psychological symptoms of dementia.

The recruitment and allocation procedures are shown in **Figure 1**. The study coordinator will consult with the treating doctors in the 12 medical institutions to screen potential eligible patients and then contact patients and carers to obtain informed consent. A screening assessment will be performed to confirm their participation eligibility, and participants who meet the inclusion criteria will complete a baseline assessment simultaneously. Subsequently, 12 medical institutions are randomly assigned to the control group (conventional intervention) and the DICE intervention group in a 1:1 ratio. In the randomization process, 12 medical institutions will be numbered, and 12 envelopes will be prepared, of which six are written on usual care and six on DICE intervention. The researchers will open random envelopes according to the coding order of the institutions, determine the grouping of an institution according to the allocation scheme in the envelope, and then the coordinator will inform the participating medical institutions of their respective grouping. Baseline and follow-up efficacy assessors will be excluded from the randomization process to ensure blinding. Study assessors will be forbidden access to the medical records from and clinical care discussion of participants. Study coordinators, treating doctors, and assessors will strictly adhere to the randomization process and blind principles to ensure the accuracy of experimental data.

### Intervention

#### AI-aided DICE algorithm

The intervention group will use the AI-aided DICE algorithm. The AI-aided DICE algorithm requires doctors to manage patients with the assistance of programs on smartphones, which includes four steps as follows:

#### Describe

Doctors will synthesize assessment results to evaluate the performance, severity, and the burden on caregivers of BPSD in patients through the AI-based application on smartphones, which names SPROUT, an AI-assisted social prescription decision system. The application provides assessment tools for doctors to understand the patient’s situation. SPROUT, with a community-centered approach, delivers four core services: mental and physical health assessment, assessment record management, clinical diagnosis management, and personalized intervention. The BPSD assessment within the mental and physical health assessment module is constructed based on the NPI-Q framework, featuring 12 domains that each integrate specialized assessment scales: the Cohen-Mansfield Agitation Inventory (CMAI) for evaluating agitation and aggressive behaviors, the Behavioral Pathology in Alzheimer’s Disease Rating Scale (BEHAVE-AD) for assessing psychotic symptoms, and the Mayo Sleep Questionnaire (MSQ) for measuring sleep-wake disorders, along with other standardized BPSD evaluation tools. This module will enable clinical intervention teams to conduct comprehensive assessments of BPSD manifestations and severity levels, thereby supporting a person-centered approach to understanding overall clinical conditions of patients. Doctors can quickly assess and describe the patients’ behavior and emotions. When completing the application questions, doctors can check patients’ scores in different behavioral and emotional dimensions and make clear the symptoms of patients.

#### Investigate

The mental and physical health assessment module of SPROUT additionally incorporates structured interview questionnaires covering basic physical condition, social participation/support, lifestyle, caregivers burden, and so on. Clinicians will utilize these instruments to conduct in-depth patient evaluations. Doctors will adopt the interview outline based on the behavioral analysis model to understand the physical situation, personality, significant life events, and interests related to the target symptoms of BPSD in patients. At the same time, other potential risk factors, like caregivers’ communication skills, living environment, and so on, will also be involved. These tools are accessed on the above application. On the application, doctors can find the clinical records of the patients and quickly locate their interested interview outline.

#### Create

Based on information from the previous two steps, SPROUT will give corresponding suggestions and provide a list of non-pharmacological treatments, including guiding caregivers to use the “Elderly Cognitive Care Toolbox,” establishing effective communication with patients, engaging patients in meaningful activities, conducting cognitive activation training at home, and recording care diaries and summarize care experiences. Doctors make treatment plans for patients according to their own clinical judgments. Then, the system will automatically generate a scheme for the caregivers.

#### Evaluate

The SPROUT system will enable clinicians to locate patients via its assessment record management module and create evaluation records at designated follow-up nodes, maintaining content consistency with the core mental and physical health assessment module. Doctors follow up with patients monthly to understand changes in their BPSD, record the implementation of individualized intervention measures, and dynamically adjust the intervention measures and methods based on symptom changes and treatment guidelines until the end of the 12-week follow-up period of the study. The patient’s clinical data will be stored and organized in the application. Thus, doctors can promptly determine the patient’s condition and handily update them.

### Control intervention

Patients in the control group receive usual care, which entails treatment based on the medical regimen physicians provide at the current research center, including pharmacological and non-pharmacological interventions. Physicians in the control group will not be required to follow the DICE approach. They may make clinical decisions based on their own experience without the need to manage AD patients with BPSD in a structured manner following the four steps of the DICE approach.

### Withdrawal criteria

All participants can withdraw from the study at any stage, regardless of providing a reason. Participants who withdraw from the study will not face discrimination or retaliation, and their medical treatment will not be affected. During the study, researchers have the authority to withdraw participants from the study based on ethical, compliance, management issues, or other reasons.

we will minimize withdrawals. Participant withdrawal from the study may occur under the following circumstances: if for medical reasons, the researcher deems it unsuitable for the participant to continue in the study; if the participant’s compliance with the protocol is poor and is judged by the researcher and project team to significantly affect the assessment of efficacy; if the participant requests to withdraw from the study; if the participant is lost to follow-up [defined as three consecutive unsuccessful attempts (not on the same day) to contact the participant for follow-up assessment or examination via phone, email, etc. All unsuccessful attempts should be documented in the study files]; if the researcher assesses that the participant poses a significant risk of harming themselves or others or if the NPI-Q agitation/aggression item score = 3 and the caregiver distress score for that item is ≥ 4; if for any reason the researcher, project team, ethics, or regulatory body requests the termination of the study.

Participants who withdraw from the study will complete early termination visits as arranged by the researcher as soon as possible; this excludes participants who withdraw informed consent or are lost to follow-up. Researchers will strive to obtain the reasons for participant withdrawal from the study, and these reasons will be documented in the case report form. Suppose a participant withdraws from the research and revokes consent for future data disclosure. In that case, further data collection may not be carried out, but the project team can retain and continue to use data collected before the withdrawal of informed consent.

To minimize loss to follow-up, we will implement the following measures:

Telephone Follow-up: We will contact the subject’s caregiver by phone to determine the reason for withdrawal and attempt to resolve any issues. Follow-up calls will continue until either the issue is resolved or the caregiver explicitly declines further participation (maximum of three attempts).
In-Person Interview (Optional): If subjects or caregivers considering withdrawal agree to discuss their decision, we will arrange a meeting with researchers and clinicians to address their concerns. Follow-up efforts will continue unless they explicitly decline further participation.

### Outcome measure

The primary outcome will be the change in score on the NPI at week 12 compared to the baseline visit. The secondary outcomes will be the change in score on the NPI at the week one and week 4 visit compared to the baseline visit and the change in score on the Caregiver Burden Inventory (CBI) at the week 1, 4, and 12 visits compared to the baseline visit. Exploratory outcomes will be the salivary cortisol level and the eye-movement index under the emotional recognition task. What’s more, during each visit, the compliance of patients and caregivers, patients’ combined medication and adverse events will be recorded. The timeline of assessments in the current study is shown in **Table 1**.

**Table 1:** Timeline of study assessment.

Measure	Screen visit	Baseline visit	Intervention visit		
	Week -1	Day 0	Week 1	Week 4	Week 12
Demographic information	×				
Medical history	×				
NPI-Q	×				
NPI		×	×	×	×
CBI		×	×	×	×
Salivary cortisol		×	×	×	×
Emotional recognition task		×	×	×	×
Care record			×	×	×
Compliance record			×	×	×
Pharmaceutic treatment record			×	×	×
Adverse events			×	×	×

NPI-Q, Neuropsychiatric Inventory Questionnaire-Informant Version; NPI, Neuropsychiatric Inventory; CBI, Caregiver Burden Inventory.

### Primary outcome

The NPI is a standard tool to evaluate 12 psychiatric behavioral symptoms in dementia patients, including delusions, hallucinations, agitation/aggression, depression, anxiety, euphoria, apathy, disinhibition, irritability, aberrant motor behavior, sleep disorder, and appetite disorders (3). According to the situation of the patient, the frequency (score range 0-4) and intensity (score range 0-3) of each item are evaluated, and the caregiver’s distress caused by each symptom (score range 0-5) is also included.

### Secondary outcomes

Except for the change in NPI score in weeks one and four and the change of distress of NPI in weeks 1, week 4, and week 12, we also adopted the CBI as the secondary outcome. CBI is a commonly used tool for assessing caregiver burden. The CBI consists of 24 items, with a score range of 0–4 for each item (15). The CBI includes five dimensions: time-dependent burden, developmental burden, physical burden, social burden, and emotional burden. Higher scores indicate a heavier burden on the caregiver.

### Exploratory outcomes

The exploratory outcomes include the salivary cortisol, which somewhat reflects the pressure level. Subjects were collected saliva by using saliva collection tubes. Since salivary cortisol exhibits a strong diurnal rhythm, we adopted the morning cortisol level (upon waking) — a commonly used exploratory outcome in related studies — as the measurement indicator (16). Therefore, in this study, saliva samples were collected immediately after waking (between 6:30 AM and 7:00 AM). Before and during collection, participants remained at rest and were not allowed to consume any liquids or food. The samples were stored at 4°C until they were sent to the laboratory within one week. Upon receipt, the saliva samples were centrifuged at 3000 rpm for 15 minutes and stored at -20°C. Salivary cortisol levels were measured using electrochemiluminescence immunoassay.

The eye-movement index under the emotional recognition task will be recorded by an eye-tracking system mounted in a VR helmet. In the emotion recognition task, participants are required to wear VR helmet and observe the movements of characters within their field of view to perceive their emotions. During data recording, the accuracy of the participants’ emotion perception as well as their pupil gaze behavior are recorded. After defining areas of interest (AOIs), the fixation points are preprocessed to calculate the number of fixations on each ROI, the duration of fixations, and the number of saccades between different AOIs, in order to understand the participants’ attention patterns (17).

### Statistical analysis and sample size

The main analysis will utilize a mixed-effects model for repeated measures (MMRM) to analyze the relative change in NPI score over time compared to baseline. In the MMRM model, the change in NPI scores relative to baseline after intervention will be used as the dependent variable, with the baseline NPI score as a covariate and the group, visit time, and interaction item, group * visit time, as fixed effects. The least squares mean, 95% confidence intervals, and *p*-value for the changes in scores before and after intervention will be estimated for each group. The least squares mean, 95% confidence intervals and *p*-value for comparing the intervention and control groups regarding the relative change in NPI scores from baseline after 12 weeks of intervention will also be estimated.

We will use a covariance analysis model to analyze the relative baseline changes in NPI scores at 12 weeks of treatment. The model will include baseline NPI scores as covariates and treatment groups as fixed effects. The least squares mean and 95% confidence intervals will be calculated for the change in values before and after treatment. The difference in the least squares means, 95% confidence intervals and *p*-values for the relative baseline NPI scores at 12 weeks of treatment between the DICE intervention group and the control group will be computed. For the primary efficacy endpoint, if NPI scores are missing at 12 weeks, the last observation carried forward (LOCF) method will be used to impute missing data for NPI scores based on the baseline value. Supportive analyses for efficacy will be performed based on the per-protocol set (PPS) using the abovementioned analysis.

The current study is a cluster RCT, with the change in total score on the NPI as the primary outcome. The plan is to recruit participants from 12 medical institutions, with six institutions assigned to the intervention group and six to the control group. Referring to the study by Bachinskaya (18), the intervention group is expected to decrease four points in the NPI total score after the intervention. In contrast, the control group is expected to have a reduction of one point. The standard deviation for both groups is assumed to be four. With a two-sided α of.05 and a power of 80.0%, assuming a sample size ratio of 1:1 between the two groups and a design effect of 0.1, the required sample size for each medical institution is calculated using PASS 15 to be 13. Therefore, the sample size for the intervention group is N1 = 6*13 = 78, and the sample size for the control group is N0 = 6*13 = 78, too, resulting in 156 participants needed for enrollment. Considering a possible dropout rate during 12-week visit window of 15.0% (19), a total of 184 participants will be recruited in the current study.

### Ethics and dissemination

This study will strictly follow the principles of medical ethics to protect the rights and privacy of the participants. Research will only be carried out with the approval of the Medical Ethics Committee. In accordance with the ethical review opinions and relevant regulations, all participants and their guardians will sign informed consent before participating in the study. We provide separate informed consent forms for patients and caregivers. For patient consent form: Both the patient and their caregiver must sign. If the patient is unable to provide a signature, only the caregiver’s signature is required. For caregiver consent form, only the caregiver’s signature is necessary. The investigator is required to sign the Protocol signature page confirming that they agree to carry out the study per these documents and all the rules and procedures in the protocol before the study and agree that the responsible unit of the project and the Ethics Committee will have access to the relevant data and records if necessary. In addition, all researchers in this study have completed the research ethics training and obtained the researcher qualification before participating.

The findings from the data analysis will be disseminated in various ways, including abstracts, posters, presentations at conferences and published manuscripts in peer-reviewed journals. We will also report the research findings to the funding body, institutes and hospitals participating in and supporting the study. Study team members will own publishing and authorship rights per the International Committee of Medical Journal Editors requirements for authorship and as described in research agreements.

### Data management

According to the Good Clinical Practice (GCP) guidelines, the project team is responsible for implementing and maintaining a quality assurance and quality control system based on corresponding Standard Operating Procedures (SOPs). The project team will conduct quality control at each data processing stage to ensure the data’s accuracy, consistency, completeness, and reliability. Additionally, the project team will invite professional Clinical Research Organizations (CROs) to conduct audits of the research process. During these audits, authorized inspectors can review all study-related documents.

This study employs an electronic data capture system for data collection and management. According to the study protocol, the data manager is responsible for drafting the technical specifications of the case report form, and the database designer constructs the electronic case report form based on these specifications. The electronic data capture system sets permissions control based on different roles.

## Discussion

The implementation of the study will hold significant implications for the management and treatment of BPSD in relevant medical institutions in China. Research teams from other countries have conducted studies on the DICE approach and have found positive effects (7, 20). However, considering the uneven distribution of medical resources in China and the inadequacy of community hospitals in diagnosing and treating BPSD, the applicability of this clinical approach in China remains unknown. Based on years of research and patient management experience, the research team has optimized the original DICE approach to make it more accessible for learning and dissemination. This study not only aims to provide scientific evidence and essential references for the optimized DICE approach in BPSD management and offers scientific solutions to the clinical challenges in geriatric psychiatry. Furthermore, it will improve healthcare services for dementia patients at various levels of medical institutions and enhance the quality of healthcare services in China.

The unique outcome of the study lies in validating the AI-aided DICE algorithm for effectively intervening in BPSD in AD patients at different levels of medical institutions. In the preliminary stage, our research team explored and optimized the application of the DICE approach combined with the AI algorithm. We have selected assessment tools to assist in describing symptoms and developed a behavioral analysis framework to guide investigations into the underlying causes of BPSD. We have completed clinical management recommendations for BPSD based on the consensus of experts in the field of neurocognitive disorders. Additionally, we have developed an “Elderly Cognitive Care Toolkit” to guide person-centered non-pharmacological interventions. Importantly, we have integrated most of the steps mentioned above into the application of smartphones. In other words, doctors don’t need extensive experience dealing with BPSD patients. When they start this application, they can conveniently and quickly manage patients with BPSD according to the specifications. AI can also make timely suggestions according to the patient’s situation so that doctors can make clinical decisions.

We pay attention to DICE, a non-pharmacological intervention, because some problems still inevitably appear in the pharmacological treatment of BPSD. The effect of pharmacological treatment may be limited. For example, a meta-analysis suggested limited efficacy of atypical antipsychotics in treating BPSD, with NPI score reductions ranging from 0.13 to 0.16 after treatment (21). Similarly, a 36-week study funded by the National Institute of Mental Health in the United States, known as CATIE-AD, targeting AD patients with psychiatric symptoms, aggression, or agitation, found no significant differences in efficacy and tolerability between different atypical antipsychotics and placebo (22). Therefore, the use of antipsychotic drugs for treating BPSD has been controversial, especially considering the potential risks of adverse reactions such as sedation, extrapyramidal symptoms, gait abnormalities, and even an increased risk of mortality for patients (23, 24).

In this research proposal, we outline how we will apply the AI-aided DICE algorithm to intervene in AD patients with BPSD, aiming to verify the effectiveness of this intervention through a cluster randomized controlled trial. However, the study still has certain limitations. Firstly, patients come from different households, each with its characteristics, and caregivers may have personalized approaches to caring for patients. Although we provide standardized DICE methods in the intervention group, it is uncertain whether caregivers can successfully implement them at home and whether care meets our requirements. We find it may be challenging to track and observe directly. Nevertheless, differences among households may influence our research results. Secondly, our primary outcome is assessed through scale evaluations. Assessors inquire with patients and their caregivers to complete the assessment. However, this behavioral measurement method may inevitably be subject to memory biases. Additionally, we are examining behavioral changes, and more than the 12-week time frame may be needed to observe significantly noticeable changes in behavior.

## Ethics statement

The study was approved by the ethics committee of Peking University Sixth Hospital (2023lunshen-No.10, 2022lunshen-No.2). All participants provided written informed consent.
